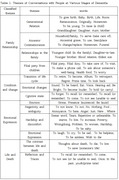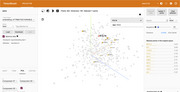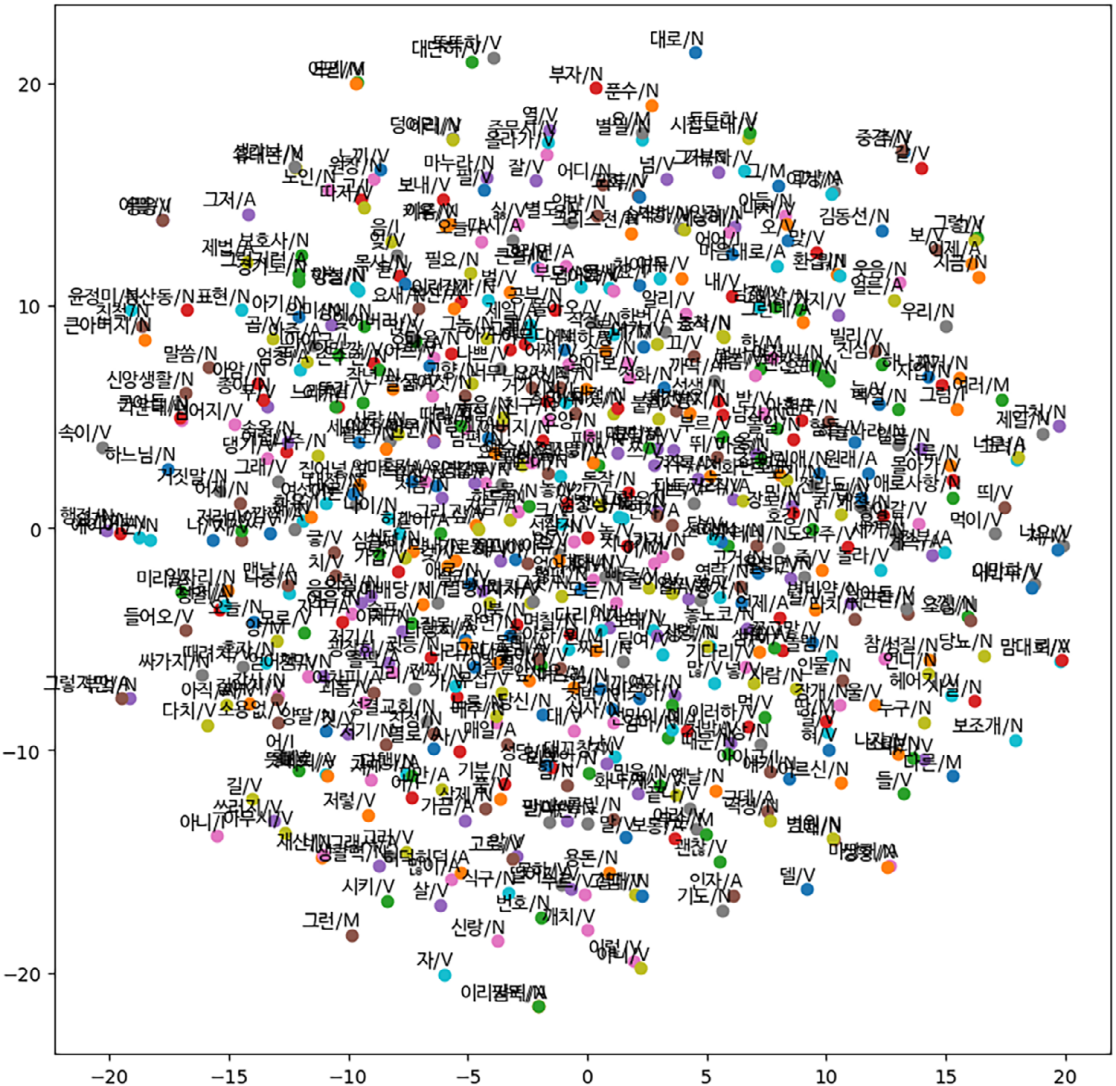# Topic Modelling Analysis of the Conversation with People at the Various Stages of Dementia

**DOI:** 10.1002/alz70858_099551

**Published:** 2025-12-24

**Authors:** Dongseon Kim

**Affiliations:** ^1^ Sookmyung University, Seoul, Daejeon, Korea, Republic of (South)

## Abstract

**Background:**

To better use of the innovative intervention such as AI speaker and social robots for the older people with cognitive difficulties, it is indispensable to discover the way and meanings within their discourse. This understanding is also a cornerstone of person‐centered care, as it enables caregivers to understand their interests and desires. This study aims to understand the experiences and needs of people with dementia through conversations with them.

**Method:**

Conversations with 108 individuals at various stages of dementia were collected over 1.5 years by three interlocutors. Participants included those with mild cognitive impairment (20.37%), early‐stage (29.6%), middle‐stage (34.3%), and severe dementia (15.7%). The dataset totalling 5,290 minutes was transcribed and organized into 1,171 episodes averaging 4.5 conversational turns. Transcriptions were analyzed using KoNLPy, Python, and BERTopic to perform word frequency, *n*‐grams, and topic modeling analyses.

**Results:**

Frequency analysis revealed the distinct linguistic characteristics of people with dementia. Among the highest frequently used 100 words, 18 pronouns and vague demonstratives such as “that,” “this,” “what,” and “just” accounted for 28.65% of total occurrences. Other frequently used words included those related to time, relationships and reminiscence. Global Maintenance Measurement which evaluate how closely each conversation aligns with a central topic showed significant differences across dementia stages: mild cognitive impairment scored 3.8, early stage 3.6, middle stage 3.2, and severe dementia 2.3 (F=5.369***). BERTopic modeling identified 30 initial topics, refined into 9 final themes, including family relationships, life and death, social interaction, identity, and agency. Word networks visually illustrated thematic connections, clarifying the contextual meanings of conversations.

**Conclusion:**

This study shows that people with dementia can engage in meaningful conversations of the various topics despite challenges such as passive responses, delusions, and repetitive speech. Analysis of nine themes derived from the conversation revealed their reflection on life and death, medical and hygienic needs, and their cognitive and sensory changes. Contrary to pathologizing views, the study highlights the identity and agency of people with dementia, showcasing their wishes to overcome the difficulties and engage in their lives. The findings stress the importance of conversation and call for technology to enhance personhood across dementia stages.